# Development of QSRR model for hydroxamic acids using PCA-GA-BP algorithm incorporated with molecular interaction-based features

**DOI:** 10.3389/fchem.2022.1056701

**Published:** 2022-11-22

**Authors:** Yiming Nie, Jia Li, Xinying Yang, Xuben Hou, Hao Fang

**Affiliations:** ^1^ Department of Medicinal Chemistry, School of Pharmaceutical Sciences, Cheeloo College of Medicine, Shandong University, Jinan, Shandong, China; ^2^ Department of Pharmaceutical Analysis, School of Pharmaceutical Sciences, Cheeloo College of Medicine, Shandong University, Jinan, Shandong, China

**Keywords:** structure retention relationships, hydroxamic acids, HPLC, molecular docking, PCA, GA-BP, double cross-validation

## Abstract

As a potent zinc chelator, hydroxamic acid has been applied in the design of inhibitors of zinc metalloenzyme, such as histone deacetylases (HDACs). A series of hydroxamic acids with HDAC inhibitory activities were subjected to the QSRR (Quantitative Structure–Retention Relationships) study. Experimental data in combination with calculated molecular descriptors were used for the development of the QSRR model. Specially, we employed PCA (principal component analysis) to accomplish dimension reduction of descriptors and utilized the principal components of compounds (16 training compounds, 4 validation compounds and 7 test compounds) to execute GA (genetic algorithm)-BP (error backpropagation) algorithm. We performed double cross-validation approach for obtaining a more convincing model. Moreover, we introduced molecular interaction-based features (molecular docking scores) as a new type of molecular descriptor to represent the interactions between analytes and the mobile phase. Our results indicated that the incorporation of molecular interaction-based features significantly improved the accuracy of the QSRR model, (R^2^ value is 0.842, RMSEP value is 0.440, and MAE value is 0.573). Our study not only developed QSRR model for the prediction of the retention time of hydroxamic acid in HPLC but also proved the feasibility of using molecular interaction-based features as molecular descriptors.

## Introduction

**FIGURE 1:**
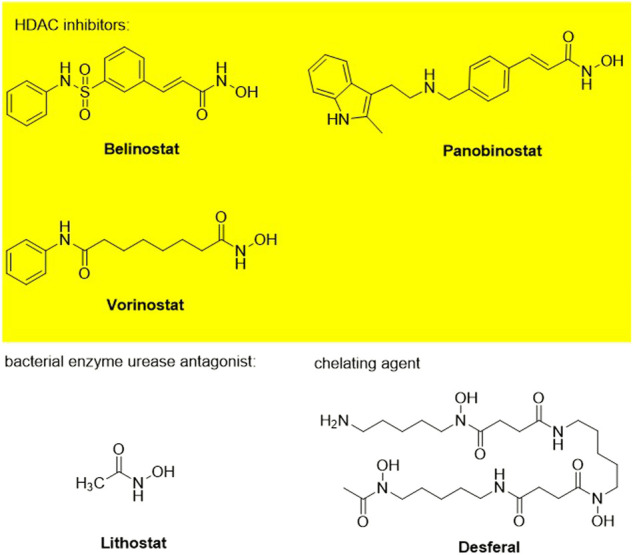
Chemical structures of hydroxamic acid-based drugs have been approved for the clinic or in clinical trails.

Hydroxamic acids have metal chelating properties. Especially, due to the high chelating power to zinc ions, hydroxamic acids are widely used as inhibitors of enzymes having a Zn^2+^ in the active site (matrix metalloproteinases (MMPs), tumor necrosis factor-alpha (TNF-α) converting enzyme, and histone deacetylase (HDAC)) (Schaal et al., 2018; Ho et al., 2020; Sanyal et al., 2022). Moreover, some drugs possessing hydroxamic acid structure, especially HDAC inhibitors, have been approved for the clinic (Figure 1) (Ho et al., 2020). However, compounds having hydroxamic acid structure usually possess poor solubility and affect their chromatography analyze of them.

Chromatography is a method by which we can obtain a lot of precise, comparable, and repeatable retention data for structure-diverse compounds. At the same experimental conditions, we can get the almost same results on analytes’ retention data. In a chromatographic system, the molecular structure of a compound determines its properties, and further, affect relative affinity for the mobile and stationary phases and, therefore, its retention characteristics (Roman, 2007; Ganesh et al., 2022). The quantitative structure-retention relationships (QSRR) have been used as a model approach to establish methods of property prediction (Roman, 2007). So the construction of the QSRR model is helpful for the chromatography of hydroxamic acids. Reliable structural descriptors are necessary for QSRR models. Here, we choose the results of the scoring functions based on experience as the new structural descriptors to regression analysis for the first time. Scoring functions are usually used to evaluate the rationality of the receptor-ligand binding mode obtained theoretically. They also can be applied to estimate the binding affinity between the receptor and ligands for molecular docking and virtual screening.

**FIGURE 2:**
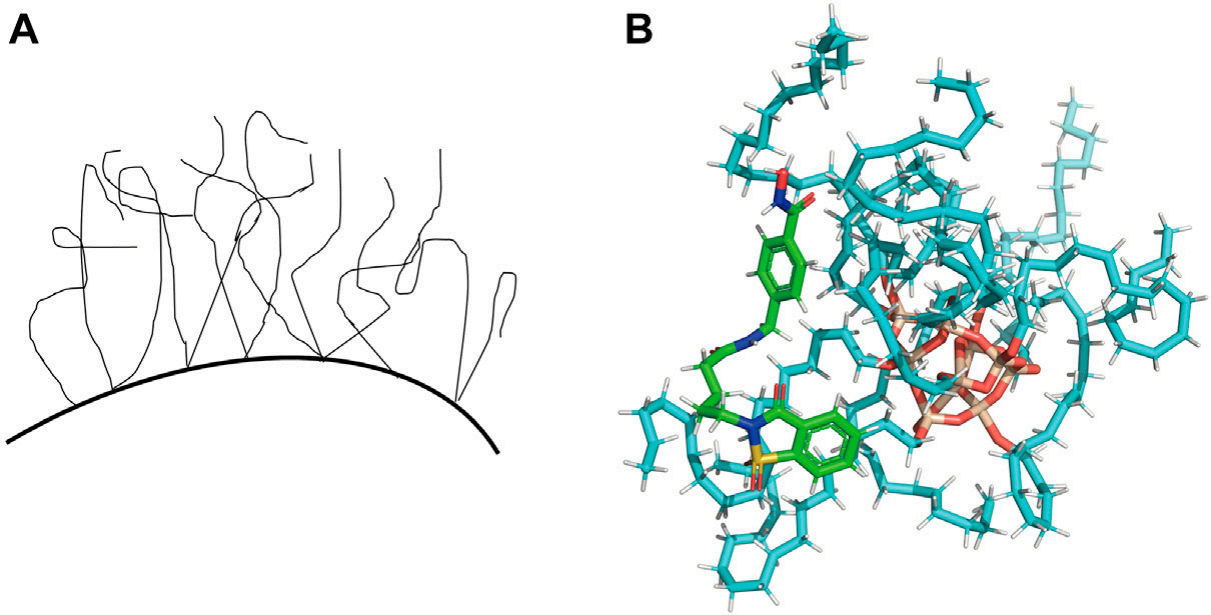
**(A)** The liquid-like configuration of ODS. **(B)** Representative docking result of one of the analytes in the ODS (C18) model.

Each analyte has its retention behavior in a chromatographic system. To great extent, the analyte’s retention behavior depends on the stationary phase of the chromatographic column and its structure. Some studies have reported the models to characterize the conformation of ODS (Octadecylsilyl) material (Figure 2A) and the interaction with solvent molecules (Ledesma and Wornat, 2000; Brambilla et al., 2007). Meanwhile, docking procedure has been used to select the best pose for each ligand to build QSPR model and docking descriptors are calculated based on the major interactions between ligand and cyclodextrin (Mirrahimi et al., 2016). So we hypothesize the material of stationary phase (ODS) and the analytes in the mobile phase as ‘receptors’ and ‘ligands’ respectively to simulate the combination between protein and ligands. Then we can get some scores that can characterize the molecular interaction-based feature and be used as descriptors through various scoring functions. We attempted to adopt the molecular modeling and docking method and consider the docking scores (molecular interaction-based features) as descriptors to characterize the interaction between the stationary phase and analytes for estimating a more reliable QSRR model.

Multiple variables can provide abundant information for the research technically. But in many cases, there may be correlations between variables, which increases the complexity of problem analysis. PCA (principal component analysis) is one of the most widely used data dimension reduction algorithms. It can eliminate noise and some unimportant information and change the variables into a few independent integrated variables which can stand for the most kinds of information existing in each original variable (Prasad et al., 2022). A certain range of information loss can save us a lot of time and cost. BP (error backpropagation) neural network is a neural network algorithm used in QSPR (quantitative structure-property relationships) research, QSAR (quantitative structure-activity relationships) research, and other models’ establishment widely and can handle complex data effectively (Luo et al., 2015; Bahmani et al., 2021; Yang et al., 2021; Xie and Xue, 2022). Therefore, we applied it in the establishment of the QSRR model. Meanwhile, the GA (genetic algorithm) is capable of optimizing the initial weight and threshold, thereby improving the robustness of the BP neural network (Zhang et al., 2019; Fang et al., 2022). And because of the wide application of hydroxamic acids in the area of HDAC inhibitors, we apply the PCA-GA-BP (Pan et al., 2007; Zhang et al., 2019; Li et al., 2020; Fang et al., 2022) neural network to establish the QSRR model to predict the retention time of hydroxamic acid-based HDAC inhibitors, for making it convenient to do HPLC and test whether molecular interaction-based features can be applied as new structure descriptors. Meanwhile, to ensure reliability and precision, we utilized double cross-validation, which contains internal (inner) and external (outer) cross-validation loops, to establish models (Roy and Arnbure, 2016).

## Experimental

### Chemicals and materials

If not specified, all chemicals were of analytical grade. The methanol was purchased from Fisher Chemical (HPLC Grade), while the formic acid was obtained from Kermel. The ultra-pure water was obtained from Heal Force SPW ultra-pure water system. All the twenty-seven analytes, including the marketed drugs or the compounds reported by our group’s former work (Wang et al., 2014; Fu et al., 2015; Liu et al., 2016), were the compounds possessing hydroxamic acid structure and were synthesized and confirmed by ourselves.

### HPLC analysis

All the compounds were tested on Agilent 1,100 system (Agilent Technologies, USA), equipped with a quaternary pump, manual injector (20 μL sample loop), and VWD detector. The column used in this study was Phenomenex Luna 5u C18 (150*4.6 mm 5micron). The mobile phase consisted of 45% aqueous phase (contains 0.1% formic acid) and 55% methanol (contains 0.1% formic acid). The flow rate was 1 ml/min and the UV detection was performed at 254 nm. The retention time of each analyte was shown in Supplementary Table S1.

### QSRR model generation

#### Calculation of the molecular descriptors

The steps of descriptor generation were as follows. First, we sketched molecular structures with ChemDraw 14.0. Then the molecules were minimized using Tripos Field (White, 1977; Motoc et al., 1986; Waltho et al., 1988; Clark et al., 1989; Purohit et al., 2020) in Sybyl-X 2.0 (SYBYL, 2012; Liang et al., 2021). The lowest energy structures were further optimized by undergoing (Turner et al., 2017) (PM7) (Stewart, 2013; Turner et al., 2017) in Molecular Orbital PACkage (MOPAC 2016) method. Then we used Schrödinger software to carry out the optimization function applying the Becke 3-parameter (exchange) with correlation by Lee Yang and Parr (B3LYP) (Becke, 1988; Lee et al., 1988; Becke, 1993; Stephens et al., 1994; Adekoya et al., 2022; Sakr et al., 2022) functional and the 6-31G (**) (Frisch et al., 1984; Adekoya et al., 2022; Sakr et al., 2022) basis set, density functional theory (DFT) (Calais, 1993; Koch and Holthausen, 2001; Adekoya et al., 2022; Sakr et al., 2022), and the standard Poisson-Boltzmann continuum solvation function (PBF) (Gilson et al., 1985; Sharp and Honig, 1990; Tomasi and Persico, 1994; Wang et al., 2017) for further geometry optimization.

The Molecular Descriptors function of Schrödinger software was applied to descriptors calculation. The molecular descriptors, Schrödinger software can calculate, contain three main categories: Topological Descriptors, QikProp Properties, and Semiempirical Properties. The Handbook of Molecular Descriptors (Roberto Todeschini and Consonni, 2000) details the calculation procedure. 273 molecular descriptors were obtained, and for the sake of minimizing subsequent problems of chance correlation, descriptors which were constant or near-constant values, less than 0.0001 concerning standard deviation, strongly correlated (descriptors with a correlation coefficient >0.90), and not available for all compounds were excluded. After the pre-reduction step, 197 molecular descriptors were obtained.

#### Molecular docking

The ODS model was manually established using Sybyl-X 2.0. All the analytes were docked against the ODS model using Surflex-Dock module (Jain, 2007; Singh et al., 2019). The representative docking result is shown in Figure 2B. For each analyte, the docked conformation with the highest was selected and 8 different types of docking scores as new descriptors, including Total_Score, Crash, Polar, D_SCORE, PMF_SCORE, G_SCORE, CHEMSCORE, GLOBAL_SCORE (Kuntz et al., 1982; Eldridge et al., 1997; Jones et al., 1997; Muegge and Martin, 1999). The docking scores of each analyte were summarized in Supplementary Table S2 in Supporting Information.

#### Dimension reduction and the construction of the QSRR model

Because the descriptors were too much to get a good QSRR model rapidly, we performed the PCA on two matrixes, one consists of the descriptors without molecular interaction-based features and the other is made up of the descriptors with molecular interaction-based features, in Matlab (Matlab, 2013) to achieve the dimension reduction of independent variables (molecular descriptors). Before that, all the descriptors had to be standardized. The principal components which can reflect 95% of the original data were chosen for the building of the QSRR model.

The matrixes we obtained through PCA were independent variables and we added the retention time of each compound as the dependent variable to form two new matrixes. The retention time of each compound was added into the matrixes consisted of principal components to form two new matrixes. Each matrix was separated into two groups randomly, of which one was the training group consisted of twenty samples and the other one was the testing group consisted of seven samples. Each line was a sample and every column except the last one was the independent variable and the last column was the dependent variable.

GA-BP was performed on these two matrixes respectively. The matrix was divided into training group (20 compounds) which was used in the inner loop for the model training and selection, and test group (7 compounds) utilized to validate the precision of model externally. In the inner loop, we utilized 5-fold cross validation which means that we separate the training group into five portions and take four portions for training and one for validation for five times. Then we compared the mean absolute error (MAE) value of these five model in the internal validation and selected the one whose MAE value is lowest to process external prediction (Roy and Arnbure, 2016). Levenberg-Marquart optimization algorithm (Raja et al., 2021) was chosen for training step in Matlab R2020a. The number of the hidden layers and hidden neurons of each layer is significant for the BP model. The addition of hidden layers can reduce the error but also can make the network complicate and increase the training time and the tendency of over-fit. A single sufficiently largely hidden layer is adequate for the approximation of most simple functions (Reed and Marks, 1999). So one hidden layer is enough and we can increase the number of hidden neurons to improve the precision. The number of hidden neurons was determined with the experimental function (1).

$$h=\sqrt{m+n}+\alpha \;\left(h<N-1,\;m<N-1\right)$$ (1)

h = the number of hidden neurons, m = the number of input nodes, n = the output nodes, *α* = adjustment constant between 1 and 10, and N = the number of training samples.

To validate the accuracy of GA-BP models built for this study, the R^2^, the root-mean-square error prediction (RMSEP) and MAE were measured for an independent set of analytes that were completely separate from the training set used in creating a QSRR model. R^2^ and RMSEP were defined as the function 2) (Alexander et al., 2015) and 3) (Žuvela et al., 2015) respectively.

$$R^{2}=1-{\sum \left(yi\left(obsd\right)-yi\left(pred\right)\right)}^{2}{/\sum (yi\left(obsd\right)-\bar{y})}^{2}$$ (2)

$$RMSEP=\sqrt[{2}]{\overset{n}{\underset{i=1}{\sum }}{\left(\frac{yi\left(obsd\right)-yi\left(pred\right)}{yi\left(obsd\right)}\right)}^{2}/n}$$ (3)

$$MAE=\frac{\overset{n}{\underset{i=1}{\sum }}\left|yi\left(pred\right)-yi\left(obsd\right)\right|}{n}$$ (4)

yi (obsd) = observed retention time, $\bar{y}$ = the mean of observed retention time, yi (pred) = predicted retention time, n = the number of analytes.

#### Applicability domain

The purpose of establishing the QSRR model is to predict the retention value of new chemical entries falling within the applicability domain (AD) of the developed model. The reliability of any QSRR model relies on the confident predictions of these new compounds based on the AD of the model, and therein lies the importance of the AD study. Therefore, we harnessed an approach which has been reported to test the compounds we used whether were suitable. If the means of the S_i_ (the corresponding standardized value for principal component *i* of one compound) values of a compound for all components in a model plus 1.28 times corresponding standard deviation (call it S_new_) is lower than 3, then there is 90% probability that the S_i_ values of that compound are lower than 3. Thus, when S_new_ value of a compound is lower than 3, then the compound can be considered to be not an X-outlier (if in the training group) or within the AD (if in the test group). (Roy et al., 2015).

## Results and discussion

### Generation of principal components

After the dimension reduction by the PCA, some components were obtained. The principal components were ranked according to the contribution rate and we selected the first few components whose cumulative contribution rate can reach 95% to form new matrixes. The matrix generated from the matrix without molecular interaction-based features was named matrix 1, the other one called matrix 2. The contribution rates of these principal components of two matrixes are shown in Table 1 and Table 2 (The principle components of each analyte of two matrixes were shown in Supplementary Tables S3, S4).

**TABLE 1:** Principal Components and their contribution rates (PCA was performed on the matrix consisting of descriptors without molecular interaction-based features).

Principal components	Contribution rates%
PC 1	47.26435108
PC 2	21.77876205
PC 3	8.443341288
PC 4	6.057161663
PC 5	4.912323874
PC 6	3.076686032
PC 7	2.121700455
PC 8	1.393171762
Total	95.0474982

**TABLE 2:** Principal Components and their contribution rates (PCA was performed on the matrix consisting of descriptors with molecular interaction-based features).

Principal components	Contribution rates%
PC′ 1	45.58673414
PC′ 2	21.67436145
PC′ 3	8.723766792
PC′ 4	6.572024529
PC′ 5	4.802069629
PC′ 6	3.051923618
PC′ 7	2.139011586
PC′ 8	1.670127367
PC′ 9	1.249293494
Total	95.4693126

According to the results of PCA performed on different matrixes, when the molecular interaction-based features are added as new descriptors, one more component was gained and the contribution rates of principal components are different. Since the PCA can reduce the correlation of variables and change the closely related variables into as few new variables as possible, these new variables are not related in pairs. It suggests the molecular interaction-based features, to a certain extent, are independent of other descriptors.

### Determination of the number of hidden neurons

With the selection range of the number of hidden neurons existing, we used each number to test on each matrix 20 frequencies respectively to find the optimum number of hidden neurons. The Regression R values measure the correlation between outputs and targets, meaning predicted retention time and observed retention time here. An R-value of 1 means a close relationship and 0 means a random relationship. We took whether the Regression R-value reaches 0.95 as a simple judgment criterion of the training model and counted the number of frequencies that R-value reaches 0.95 when the different number of hidden neurons was chosen to run. The results are shown in Table 3 and Table 4.

**TABLE 3:** The frequencies that R-value reaches 0.95 (performing on the training group of matrix 1).

h	The frequencies that R-value reaches 0.95				nr
	T_1_	V	T_2_	A	
4	5	5	6	0	0
5	5	5	3	2	0
6	12	4	3	0	0
7	3	6	6	0	0
8	8	10	5	0	0
9	3	6	1	0	0
10	8	6	5	0	0
11	6	10	4	1	1
12	6	7	6	0	0
13	11	8	2	1	0

h is the number of hidden neurons. T_1_ is T1 Set. V is the V Set. T_2_ is the T2 Set. A is the All Set. nr. is the frequencies that R values reach 0.95 at the same time. (The names of four sets were given by algorithm contained in MATLAB automatically).

**TABLE 4:** The frequencies that R-value reaches 0.95 (performing on the training group of matrix 2).

h	The frequencies that R-value reaches 0.95				nr
	T_1_	V	T_2_	A	
4	3	7	4	1	0
5	2	7	6	1	1
6	12	7	4	2	1
7	6	10	6	1	0
8	5	8	2	1	1
9	8	10	7	0	0
10	7	6	1	1	0
11	7	6	3	2	1
12	11	11	5	1	1
13	7	3	8	1	0

h is the number of hidden neurons. T_1_ is T1 Set. V is the V Set. T_2_ is the T2 Test Set. A is the All Set. nr. is the frequencies that R values reach 0.95 at the same time. (The names of four sets were given by algorithm contained in MATLAB automatically).

According to the results above, the frequencies that R-value reaches 0.95 of the All Set have been improved apparently when we added the molecular interaction-based features as molecular descriptors, which suggested they are beneficial for the accuracy of QSRR models. Meanwhile, the frequencies that R values of four sets reach 0.95 at the same time are improved as well. Through the running with different numbers of hidden neurons on different matrixes, we decided to choose 11 hidden neurons to perform the GA-BP algorithm. Because the R values of Training Set, Validation Set, Test Set, and All Set (for the avoidance of the conflict of names, we renamed them T1 set, V set and T2 set), which were assigned randomly by the Levenberg-Marquart optimization algorithm, can reach 0.95 at the same time when we took eleven hidden neurons to perform the algorithm on the training group of matrix 1 and 2 (After the PCA, the matrix generated from the matrix without molecular interaction-based features named matrix 1, the other one called matrix 2).

### The establishment of the QSRR model

We took 11 hidden neurons and a double cross-validation approach to run GA-BP on the training groups of matrixes 1 and 2, and due to the random generation of weights initialization threshold, we take the same validation set to modelling 10 times respectively, then calculated the average of each training group (Table 5). Comparing the data shown in Table 5, we found that the MAE values of training groups whose principal components related to docking scores are mostly better than those without docking scores.

**TABLE 5:** Selection of training models.

Matrix	k^th^ fold	MAE of each time										Average of MAE
		1	2	3	4	5	6	7	8	9	10	
1	k1	2.6385	0.5536	1.5793	2.6044	4.8188	6.0193	2.501	1.9305	3.6009	5.6812	3.19275
	k2	3.2298	3.673	4.0849	3.501	4.63	4.0765	4.0895	4.092	3.7948	4.5111	3.96826
	k3	2.2915	2.2608	1.7267	2.3488	2.4065	2.2028	3.1336	2.853	2.3739	2.2169	2.38145
	k4	3.7133	3.6514	1.6232	5.1061	1.8693	5.9359	4.5440	4.6063	2.0027	3.9845	3.703671
	k5	2.9817	4.4447	2.6844	3.2713	2.4513	4.9758	1.4086	3.158	2.4287	2.6385	3.0443
2	k1′	2.1501	2.6904	2.3996	3.408	4.8782	1.8289	2.0223	3.0861	3.2268	2.6011	2.82915
	k2′	3.8125	3.0321	3.1707	3.9484	1.9685	3.6707	3.8672	2.2259	4.965	2.3368	3.29978
	k3′	2.0024	2.7605	3.0042	3.2663	2.4173	2.8182	1.9522	2.6188	0.8307	1.4076	2.30782
	k4′	1.9059	3.5847	1.8069	3.0382	1.9059	3.8669	0.9266	1.8069	3.0382	3.0382	2.49184
	k5′	4.3536	3.5158	4.0454	1.1736	1.63	4.085	3.2323	5.0584	1.3107	0.822	2.92268

k^th^ fold means which one of the five portions was taken as validation test.

**FIGURE 3:**
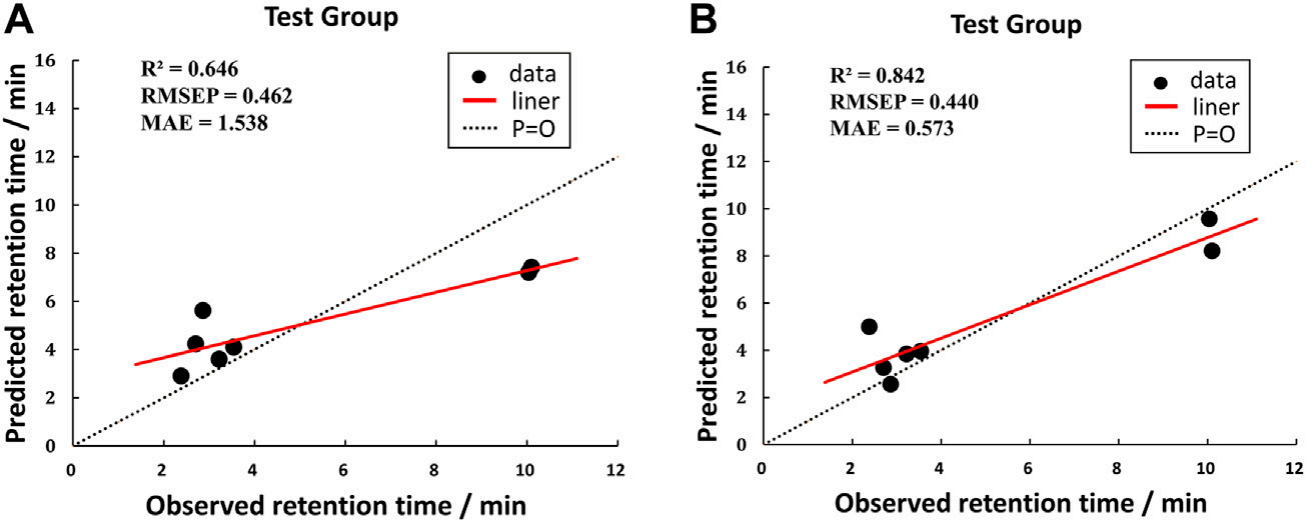
Comparison of test results of two matrixes. [**(A)** was the test group of matrix 1, and **(B)** was the test group of matrix 2].

According to the averages of MAE values, we chose the third portion as the validation set in the inner loop of matrices 1 and 2. Then we used a test group to perform external prediction. The results are shown in Figure 3 which were the linear relationships of the predicted retention time obtained by using training models together with measured retention time and Table 6. The R^2^ values of both test groups are greater than 0.6, suggesting the models are not rejected irrespective of the absolute error (Alexander et al., 2015). But when we put molecular interaction-based features into running, the RMSEP value had no apparent improvement, which has areas for further optimism. However, the MAE value becomes better. It manifested the molecular interaction-based features as an independent category of descriptors that were helpful to the establishment of the QSRR model to predict the retention time of hydroxamic acid.

**TABLE 6:** Prediction of the retention time of compounds in the testing set.

Analytes	Predicted retention time/min (model 1)	Predicted retention time 2/min (model 2)	Observed retention time/min
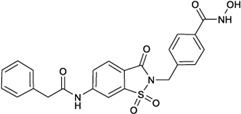	7.200123	9.573565	10.04
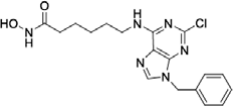	7.421031	8.218716	10.1
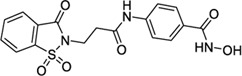	2.909212	5.000308	2.38
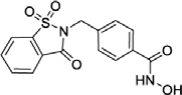	5.623486	2.573642	2.86
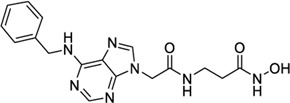	4.237541	3.273094	2.7
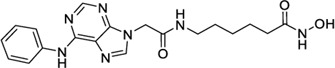	4.096308	3.965852	3.54
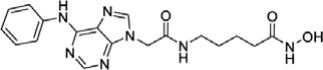	3.606235	3.847673	3.22

### Applicability domain

**FIGURE 4:**
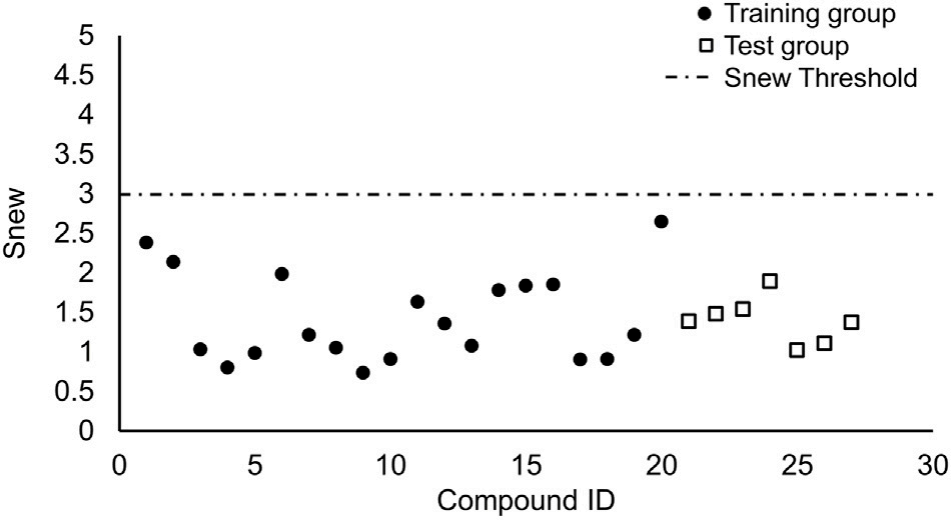
Applicability domain.

We performed the reported program to calculate the Snew values of our compounds. The result was shown in Figure 4. The Snew values of all the compounds we used are lower than 3, which means they are suitable for the model and not X-outlier (if in the training group) or within the AD (if in the test group).

### Detection of systematic error in predictions

For the determination of the quality of QSRR-derived predictions, we calculated the following five values: ratio of the number of positive and negative errors (NPE/NNE), absolute (ABS) of average error (AE) with mean absolute error (MAE), mean positive error (MPE) with mean negative error (MNE). The data was shown in Table 7.

**TABLE 7:** Detection of systematic error in predictions.

QSRR model	NPE	NNE	MPE	MNE	MAE	AE	NPE/NNE or NNE/NPE	ABS(MPE/MNE)	MAE-ABS(AE)	R^2^ (*i* ^th^ vs. (i-1)^th^ residuals)	R^2^ (Y pred vs. residuals)
model 1	3	4	0.75	-0.92	1.53	0.04	0.75/1.33	0.81912	1.501264	0.0582	0.2099
model 2	3	4	1.06	-0.89	0.57	0.23	0.75/1.33	1.209227	0.342687	0.0302	0.4208

Recording to the reported criterions: 1) NPE/NNE >5 or NNE/NPE >5; 2) ABS(MPE/MNE) > 2 or ABS(MNE/MPE) > 2; 3) MAE - ABS(AE) < 0.5×MAE; 4) R^2^ (*i* ^th^ vs. (i-1)^th^ residuals) > 0.5 for residuals sorted on Y_pred_; 5) R^2^(Y vs. residuals) > 0.5 [57], even if our models are not satisfy, in the future work, we need more analytes to improve, due to not satisfying the standard of number (at least 10).

### PLS using docking scores

**FIGURE 5:**
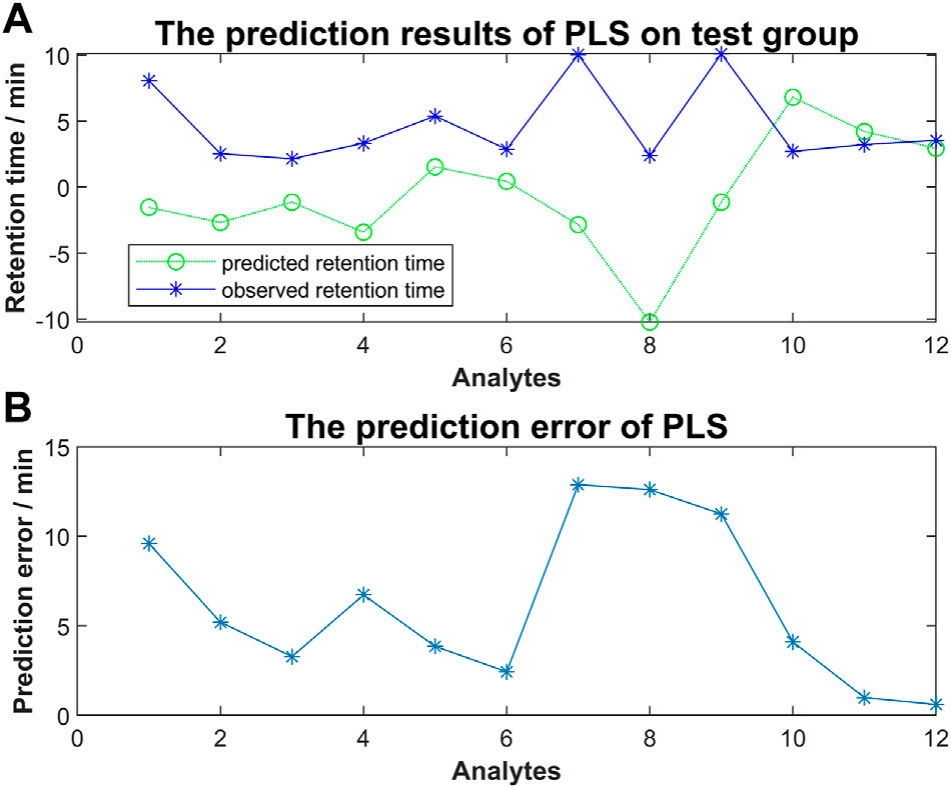
**(A)** The predicted retention time obtained by PLS method vs. observed retention time; **(B)** The prediction error of PLS for each analyte of the test group.

To investigate the relationship between molecular interaction-based features and retention time of compounds, we used molecular docking scores alone to run the PLS algorism in Matlab. Fifteen analytes were used as a training group to generate the coefficients of each type of docking score and the constant term. The simulation training and predicting of the test group were following (Figure 5). We found that a remarkable error existed between the predicted retention time and observed retention time. Our results suggested the regression using molecular interaction-based features alone to construct the QSRR model is unfeasible.

### Analyze of the relevance between molecular interaction-based features and retention time

**FIGURE 6:**
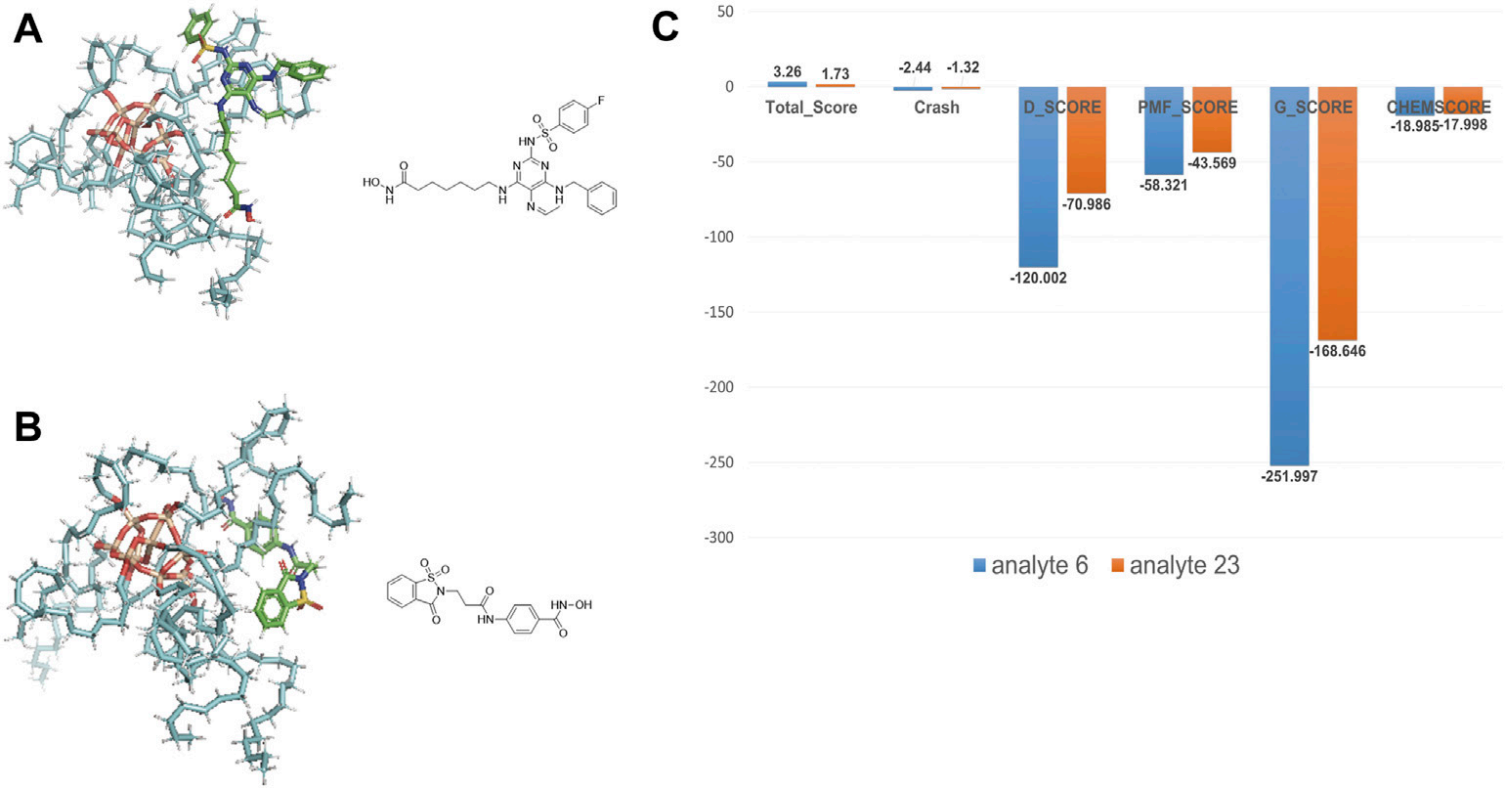
**(A)** The docking result of analyte 6. **(B)** The docking result of analyte 23. **(C)** Comparison of molecular docking scores (molecular interaction-based features) of analyte 6 and 23.

We took the molecular docking results of two analytes (analyte 6 and 23 are shown in Supplementary Table S1 and Figure 6), whose retention time is notably different (14.86 min *vs* 2.38 min), to investigate the relevance. The docking results are shown in Figures 6A,B. Obviously, analyte 6 could occupy larger area of ODS surface than analyte 23. As expected, the docking scores of analyte 6 are larger than that of analyte 23 (Figure 6C). Therefore, if the analytes could occupy more area of the material, it could generate more interaction force between these two things and have better docking result (larger minus) and large retention values. So, these molecular interaction-based features could be helpful to the prediction of analytes and improve the precision of QSRR models.

## Conclusion

The PCA-GA-BP method was employed to establish QSRR models for hydroxamic acids and the double cross-validation approach using internal 5-fold cross validation guaranteed the reliability of training model and exploited finite training compounds sufficiently. The interaction between analytes and solid-phase materials was measured using molecular docking scores, which were introduced as new features in the QSRR model. These features could manifest the strength of interaction between analytes and solid-phase material (At least in the docking of drug molecules and proteins). As a new type of molecular descriptors, molecular interaction-based features (docking scores) could contribute to the dimension reduction, selection of hidden neurons, model selection and prediction of retention time. Our QSRR model could be used to predict the retention time of hydroxamic acids.

## Data Availability

The original contributions presented in the study are included in the article/Supplementary Materials, further inquiries can be directed to the corresponding authors.
