# Supplementary material for: Development of QSRR model for hydroxamic acids using PCA-GA-BP algorithm incorporated with molecular interaction-based features
Source: Front Chem. 2022 Nov 22;10:1056701. doi: 10.3389/fchem.2022.1056701 (PMC9722961; doi:10.3389/fchem.2022.1056701)
Supplement: Supplementary file 1 [file DataSheet1.pdf]

Development of QSRR model for hydroxamic acids using PCA-GA-BP algorithm incorporated with molecular interaction-based features

Yiming Nie[a], Jia Li[b], Xinying Yang*[b], Xuben Hou*[a] and Hao Fang*[a]

[a]Department of Medicinal Chemistry, School of Pharmaceutical Sciences, Cheeloo College of Medicine, Shandong University, Jinan, Shandong 250012, China

[b]Department of Pharmaceutical Analysis, School of Pharmaceutical Sciences, Cheeloo College of Medicine, Shandong University, Jinan, Shandong 250012, China

CONTENTS

Table S1. The retention time of each analyte. 1

Table S2. The docking scores of each analyte. 6

Table S3. The principle components of matrix 1. 7

Table S4. The principle components of matrix 2. 8

* Corresponding Authors:

Phone/fax: +86-531-8838-2010 *E-mail address:* xinyingyang@sdu.edu.cn (Xinying Yang),

Phone/fax: +86-531-8838-0720 *E-mail address:* hxb@sdu.edu.cn (Xuben Hou),

Phone/fax: +86-531-8838-1168 *E-mail address:* haofangcn@sdu.edu.cn (Hao Fang).

Table S1. The retention time of each analyte.

Compound NO.	Structure	retention time / min
1	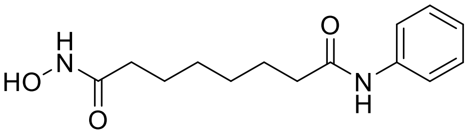	3.6
2	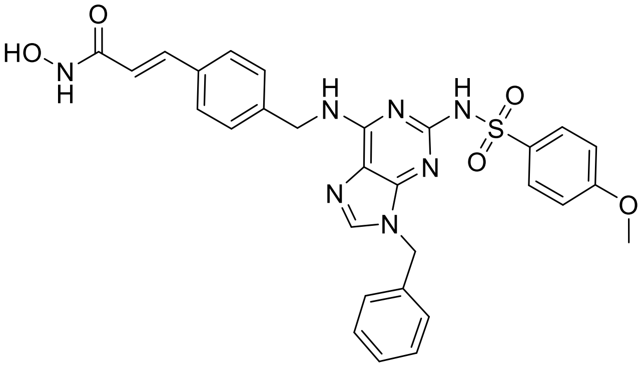	8.77
3	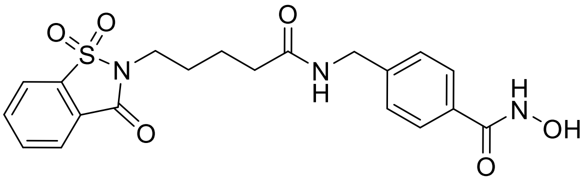	2.71
4	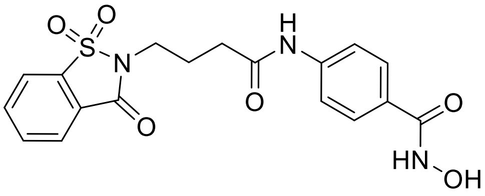	2.86
5	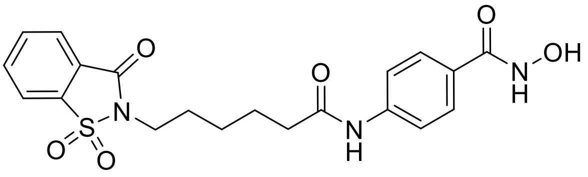	4.23

6	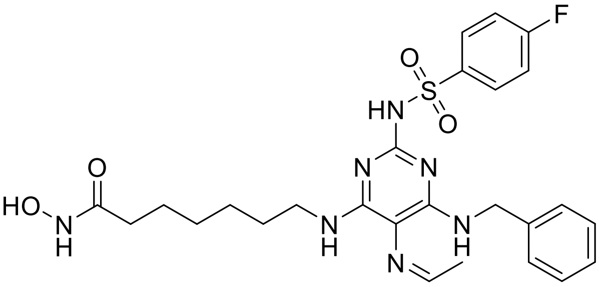	14.86
7	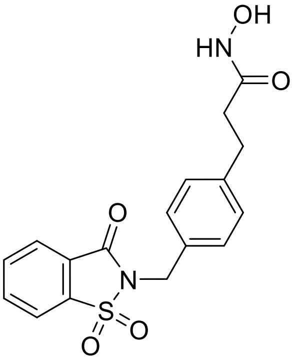	4.3
8	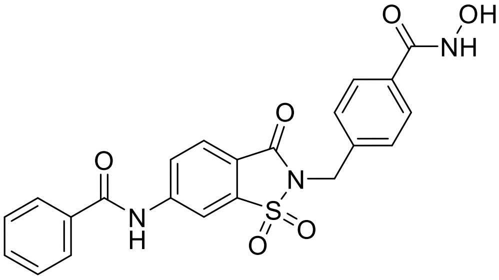	9.48
9	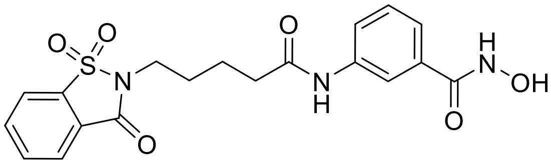	3.38
10	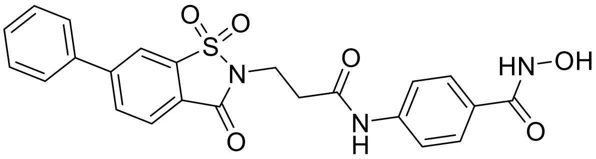	10.5

11	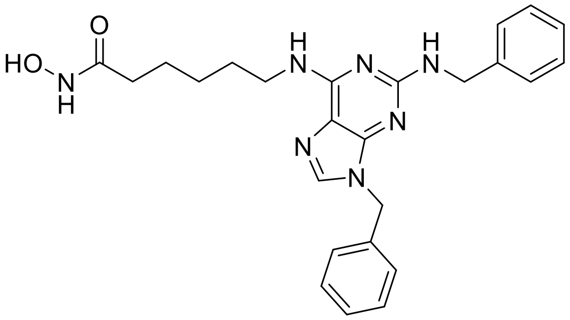	6.6
12	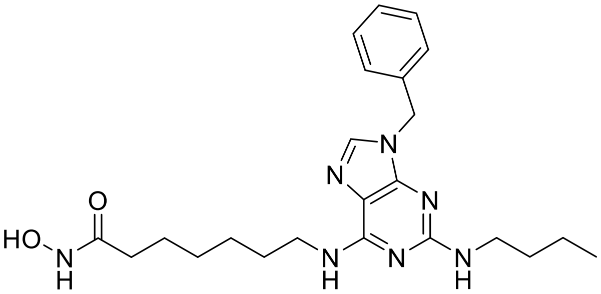	7.36
13	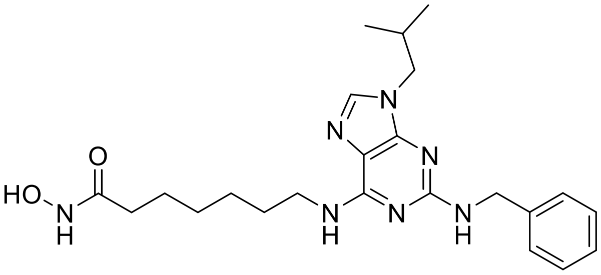	6.33
14	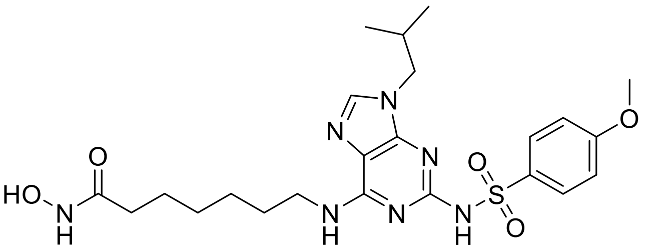	8.52
15	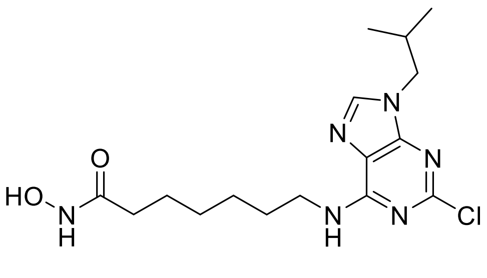	12.08

16	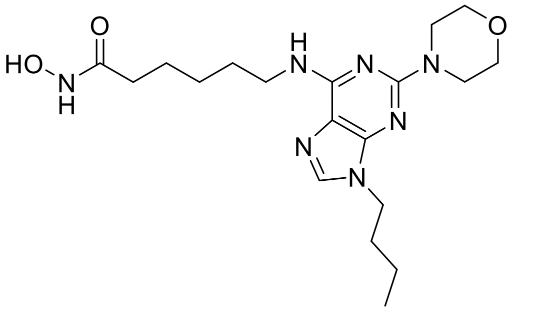	8.06
17	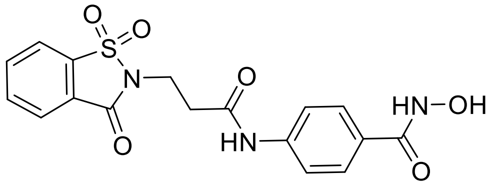	2.53
18	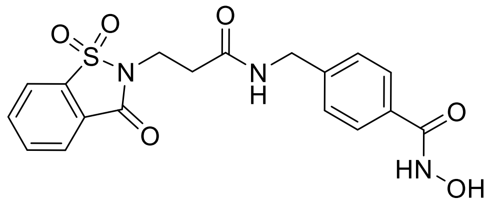	2.14
19	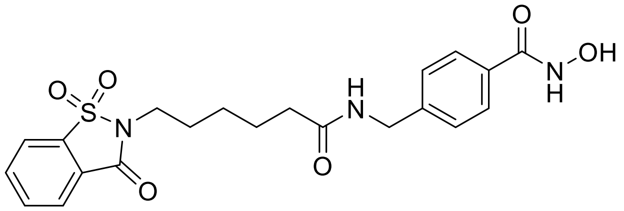	3.32
20	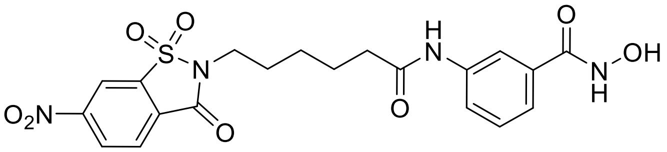	5.38
21	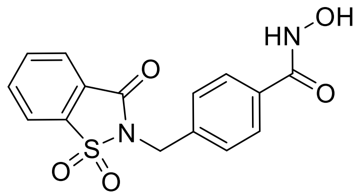	2.86

22	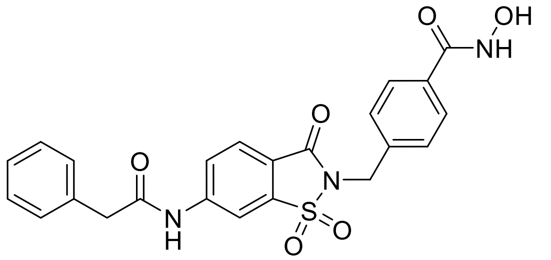	10.04
23	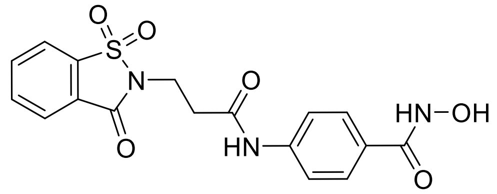	2.38
24	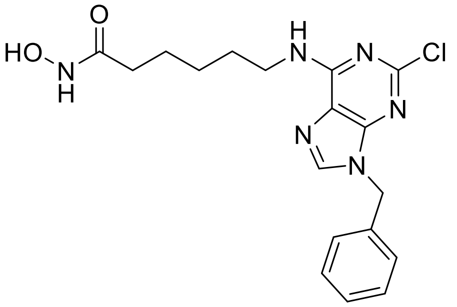	10.1
25	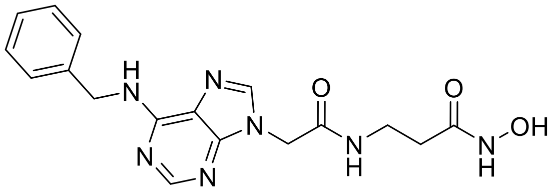	2.7
26	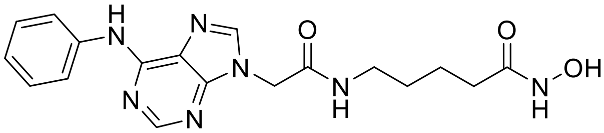	3.22
27	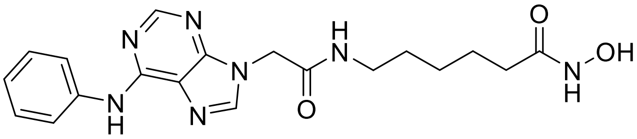	3.54

Table S2. The docking scores of each analyte.

Compound NO.	Total_Score	Crash	D_SCORE	PMF_SCORE	G_SCORE	CHEMSCORE
1	3.04	-1.29	-72.625	-36.356	-126.806	-16.212
2	1.5	-0.94	-83.299	-54.325	-137.703	-19.715
3	2.45	-1.38	-91.347	-42.177	-167.772	-17.575
4	3.51	-1.18	-87.542	-44.06	-169.457	-20.404
5	2.8	-2.1	-101.124	-46.507	-209.161	-21.027
6	3.26	-2.44	-120.022	-58.321	-251.997	-18.985
7	2.63	-0.67	-79.73	-42.222	-161.64	-16.898
8	1.66	-1.07	-79.731	-36.845	-111.582	-20.181
9	3.21	-1.26	-86.354	-33.603	-161.909	-16.822
10	3.11	-1.62	-97.64	-37.904	-210.336	-20.227
11	4.16	-1.38	-96.107	-51.894	-194.589	-15.782
12	3.69	-2.08	-103.423	-62.16	-222.057	-16.242
13	4.35	-1.95	-103.306	-58.944	-211.189	-14.623
14	3.94	-1.41	-96.857	-43.467	-198.025	-12.939
15	3.04	-1.26	-81.698	-40.866	-178.101	-11.344
16	3.96	-1.2	-92.128	-49.257	-202.407	-14.046
17	2.77	-0.88	-77.205	-43.882	-160.972	-16.714
18	2.99	-1.18	-83.386	-32.969	-150.993	-14.744
19	3.45	-1.5	-100.604	-49.717	-179.573	-20.229
20	2.33	-1.35	-90.196	-45.506	-174.371	-16.632
21	1.63	-0.76	-73.083	-41.787	-144.782	-16.502
22	2.6	-1.1	-90.244	-35.359	-175.682	-21.791
23	2.97	-0.92	-87.896	-62.003	-162.12	-28.166
24	3.1	-1.02	-89.107	-59.208	-153.547	-14.248
25	2.72	-1.25	-83.762	-43.364	-171.507	-5.438
26	2.86	-0.88	-84.902	-48.705	-176.279	-8.387
27	3.9	-2.02	-101.804	-53.741	-200.029	-10.295

Table S3. The principle components of matrix 1.

Compound NO.	PC1	PC2	PC3	PC4	PC5	PC6	PC7	PC8
1	-24.64	-6.07	7.96	-0.17	-9.43	-3.20	-0.85	0.56
2	25.20	-4.65	-2.59	-3.17	-2.96	0.36	-0.42	2.00
3	1.71	3.97	2.22	2.55	1.45	-2.59	2.52	0.74
4	-1.73	6.54	0.54	0.96	0.32	0.00	1.40	-0.28
5	1.95	4.19	2.54	1.89	1.72	-2.99	2.08	0.84
6	14.85	-7.31	7.50	-0.91	-5.66	3.22	-0.25	-0.54
7	-7.95	7.12	-0.84	-5.48	0.14	0.67	-0.86	0.96
8	7.06	6.50	-3.23	-2.59	-2.15	0.27	-0.16	0.34
9	-0.07	5.58	1.72	0.95	1.15	-1.62	1.13	-0.80
10	8.90	4.96	-2.25	-1.28	-1.27	-1.52	0.05	0.28
11	4.87	-9.09	-6.15	-2.97	-0.68	-3.94	-0.97	-1.19
12	-0.15	-10.08	0.29	-2.49	2.93	-2.96	0.08	-2.05
13	-0.05	-8.88	0.53	-2.10	2.77	-0.80	-0.24	-0.47
14	10.17	-4.24	7.85	-0.29	2.08	5.57	2.06	-0.46
15	-12.84	-4.75	3.83	-3.23	6.86	2.88	-0.83	3.77
16	-4.70	-6.58	-0.19	-1.88	3.74	0.10	0.77	-4.90
17	-3.65	7.38	-0.73	0.36	-0.49	1.66	0.68	-0.36
18	-2.03	6.31	0.17	1.64	0.11	0.36	1.50	-0.66
19	3.62	2.69	2.95	3.24	1.80	-4.04	2.57	0.86
20	7.36	5.28	4.70	6.75	3.04	-1.03	-8.38	-0.75
21	-11.36	9.87	-2.70	-5.61	-1.45	3.26	-1.42	-2.07
22	9.13	5.20	-2.28	-1.96	-1.79	-1.10	-0.69	0.66
23	-3.43	7.62	-0.54	0.55	-0.47	1.47	0.58	-0.58
24	-7.31	-5.35	-4.01	-2.90	1.99	-0.89	-1.37	3.33
25	-6.73	-4.02	-6.52	4.68	-1.53	2.17	-0.79	-0.50
26	-4.91	-5.41	-5.71	6.47	-1.25	2.86	0.82	0.25
27	-3.25	-6.77	-5.05	6.99	-0.95	1.86	0.99	1.01

Table S4. The principle components of matrix 2.

Compound NO.	PC'1	PC'2	PC'3	PC'4	PC'5	PC'6	PC'7	PC'8	PC'9
1	-24.75	-5.42	-6.61	3.92	-9.83	-2.62	-1.35	1.75	-1.13
2	24.77	-4.01	3.97	2.55	-2.91	1.61	-1.35	4.93	-1.93
3	1.62	3.65	-3.50	-2.46	1.02	-2.37	1.96	1.71	-0.20
4	-1.81	6.35	-1.60	-1.35	0.43	-0.26	1.63	-0.36	0.08
5	2.02	3.56	-3.92	-1.75	1.55	-3.27	1.93	0.71	0.70
6	14.93	-8.11	-6.44	3.83	-5.38	2.51	0.78	-3.55	3.06
7	-8.12	7.63	1.46	3.98	1.04	1.40	-0.47	1.36	-0.29
8	6.76	7.20	3.34	1.10	-1.86	0.78	0.00	0.92	0.43
9	-0.23	5.42	-2.75	-1.21	1.04	-1.41	1.03	0.51	-1.25
10	8.79	4.91	1.80	0.22	-0.77	-1.71	0.62	-0.85	1.28
11	4.58	-8.65	7.05	1.59	-0.11	-3.87	-0.89	-0.28	-0.68
12	-0.18	-10.17	0.68	2.77	3.20	-3.35	-0.03	-1.80	-0.49
13	-0.14	-9.13	0.19	2.19	3.09	-1.12	0.02	-1.25	0.74
14	10.02	-4.88	-7.34	2.00	1.91	5.64	2.08	0.27	-2.09
15	-12.98	-4.63	-3.07	3.17	6.86	3.43	-1.16	2.55	2.78
16	-4.90	-6.53	0.74	1.58	3.89	-0.13	0.75	-2.30	-4.57
17	-3.81	7.36	-0.14	-1.25	-0.28	1.65	0.94	-0.32	0.31
18	-2.24	6.25	-1.37	-2.24	-0.01	0.49	1.60	-0.04	-0.70
19	3.60	2.08	-4.30	-2.65	1.37	-4.22	2.02	1.00	0.52
20	7.25	4.52	-6.66	-5.70	1.69	-0.47	-8.71	-1.57	-0.54
21	-11.55	10.66	3.29	3.76	-0.47	3.62	-0.52	-1.97	-0.56
22	8.93	5.55	2.19	0.85	-1.41	-0.82	-0.33	0.25	0.90
23	0.68	8.15	6.09	5.33	-0.70	-1.17	-0.51	-2.98	0.31
24	-7.52	-4.94	4.70	1.46	2.32	-0.48	-1.66	2.55	2.67
25	-7.06	-4.01	5.08	-6.40	-2.05	2.34	-0.55	-0.15	-0.88
26	-5.24	-5.45	4.05	-7.55	-2.01	2.77	0.81	0.03	-0.18
27	-3.40	-7.36	3.07	-7.74	-1.60	1.03	1.38	-1.10	1.71
